# Oxygen glucose deprivation/re-oxygenation-induced neuronal cell death is associated with Lnc-D63785 m6A methylation and miR-422a accumulation

**DOI:** 10.1038/s41419-020-03021-8

**Published:** 2020-09-30

**Authors:** Shu Xu, Ya Li, Ju-ping Chen, Da-Zhuang Li, Qin Jiang, Ting Wu, Xiao-zhong Zhou

**Affiliations:** 1 Department of Neurology, the Affiliated Zhangjiagang Hospital of Soochow University, Suzhou, China; 2 The Central Lab, North District, Suzhou Municipal Hospital Affiliated to Nanjing Medical University, Suzhou, China; 3 Department of Neurology, Changshu Hospital Affiliated to Nanjing University of Chinese Medicine, Changshu, China; 4 Department of Orthopedics, the Second Affiliated Hospital of Soochow University, Suzhou, China; 5 The Fourth School of Clinical Medicine, The Affiliated Eye Hospital, Nanjing Medical University, Nanjing, China; 6 Department of Neurology, the First Affiliated Hospital of Nanjing Medical University, Nanjing, China

**Keywords:** Neurochemistry, Apoptosis

## Abstract

Oxygen glucose deprivation/re-oxygenation (OGD/R) induces neuronal injury via mechanisms that are believed to mimic the pathways associated with brain ischemia. In SH-SY5Y cells and primary murine neurons, we report that OGD/R induces the accumulation of the microRNA miR-422a, leading to downregulation of miR-422a targets *myocyte enhancer factor-2D* (*MEF2D*) and *mitogen-activated protein kinase kinase 6* (*MAPKK6*). Ectopic miR-422a inhibition attenuated OGD/R-induced cell death and apoptosis, whereas overexpression of miR-422a induced significant neuronal cell apoptosis. In addition, OGD/R decreased the expression of the long non-coding RNA D63785 (Lnc-D63785) to regulate miR-422a accumulation. Lnc-D63785 directly associated with miR-422a and overexpression of Lnc-D63785 reversed OGD/R-induced miR-422a accumulation and neuronal cell death. OGD/R downregulated Lnc-D63785 expression through increased methyltransferase-like protein 3 (METTL3)-dependent Lnc-D63785 m6A methylation. Conversely METTL3 shRNA reversed OGD/R-induced Lnc-D63785 m6A methylation to decrease miR-422a accumulation. Together, Lnc-D63785 m6A methylation by OGD/R causes miR-422a accumulation and neuronal cell apoptosis.

## Introduction

Ischemic stroke is a leading cause of human morbidity and mortality around the world^1,2^. As the prevalence of stroke rises more effective treatment strategies are urgently required^2–4^. The main pathogenesis of stroke, ischemia-reperfusion, induces significant oxidative injury to surrounding neurons^5,6^, which can be mimicked in vitro by an oxygen and glucose deprivation (OGD)/re-oxygenation (OGD/R) procedure applied to cultured neurons^7–10^.

MicroRNAs (miRs) are a class of non-coding 21–25 nt mRNA-interfering molecules^11,12^, that regulate the expression of target genes by binding to their 3′-UTR (3′-untranslated region)^11,12^. miR dysregulation is detected in cerebral pathogenesis^13–15^, and provide biomarkers in the diagnosis and prognosis of cerebral diseases^13–15^. Circulating stroke-associated miR profiles reflect temporal progression and specific etiologies of ischemic stroke^14,15^. The brain-enriched microRNA-422a (miR-422a) is upregulated in acute ischemic stroke, independent of age, severity, or confounding metabolic complications^14^. In the acute phase of stroke plasma miR-422a is significantly increased, and then downregulated in the sub-acute phase^16^. Similarly, following ischemia-reperfusion, miR-422a is downregulated in PC12 cells^17^. Here we explore whether OGD/R stimulation can affect miR-422a expression, and examine its potential functions in mediating OGD/R-induced neuronal cell death.

N6-methyladenosine (m6A) modification is the most abundant internal methylation of RNA transcripts, required for RNA processing, stabilization, and various biological functions^18^. The writer complex, that includes the methyltransferase enzymes methyltransferase-like protein 3 (METTL3), METTL14, and WTAP, catalyzes m6A modification^19^. Conversely, m6A modification is removed by the demethylases (erasers) FTO and ALKBH5^19^. Recent in vivo and in vitro studies suggest that m6A modification is involved in the mechanism of brain ischemia-reperfusion injury^19^, and indicate that inhibition of m6A methylation can protect neurons against ischemia-reperfusion injury^19^. m6A modifications regulate the function and stabilization of LncRNAs by providing a binding site for the m6A reader proteins or by changing the structure of the local RNA^20–22^. We here identify that LncRNA D63785 (Lnc-D63785) m6A methylation and downregulation is the primary cause of miR-422a accumulation in OGD/R-treated neuronal cells.

## Materials and methods

### Reagents, antibodies, and chemicals

Puromycin, polybrene, and cell culture reagents were purchased from Sigma-Aldrich (St. Louis, MO). Antibodies for MAPKK6 (#8550); MEF2D (#56830), Bcl-w (#2724), cleaved caspase-3 (#9664); cleaved-poly (ADP-ribose) polymerase (PARP) (#5625), caspase-3 (#14220), PARP (#9532), methyltransferase-like protein 3 (METTL3#86132), and GAPDH (#2148) were obtained from Cell Signaling Tech (Shanghai, China). All the sequences, primers, viral constructs, plasmids and shRNAs were designed, sequence-verified and provided by Shanghai Genechem Co. (Shanghai, China).

### Cell culture

The neuronal cells derived from SH-SY5Y neuroblastoma cells, obtained from the Cell Bank of Shanghai Institute of Biological Science (Shanghai, China), were cultured in DMEM plus 10% fetal bovine serum. For neuronal differentiation, SH-SY5Y cells were cultured in the BDNF plus glutamine medium (serum free) as previously-described^23^. After differentiation, over 95% of cells were neuronal cells. The SH-SY5Y cells were subjected to mycoplasma and microbial contamination examination every 3–4 months. Authentication by STR profiling, population doubling time, and morphology were routinely confirmed as well to verify the genotype. The primary murine neurons were provided by Dr. Di^23–25^, and cultured by the previously-described protocols^23^. At day-10 (DIV), over 95% of cells were cortical neurons. The study was approved by the Ethics Committee and IACUC committee of Soochow University.

### OGD/re-oxygenation

We utilized a previously-described OGD/R procedure^7,24^. Briefly, neuronal cells were initially placed in an airtight chamber, equilibrated for 15 min with a continuous flux of gas (95% N_2_/5% CO_2_). The chamber was sealed and placed in an incubator for additional 4 h of OGD. Neuronal cells were then re-oxygenated (OGD/R) for applied time periods. “Mock” cells were placed in norm-oxygenated DMEM containing glucose.

### Cell counting kit-8 (CCK-8)

Differentiated SH-SY5Y cells were seeded into 96-well tissue-culture plates (at 3 × 10^4^ cells/cm^2^). Following treatments, the viability was measured by a CCK-8 kit (Dojindo Molecular Technologies, Gaithersburg, MD). CCK-8 optical density (OD) values were recorded at the wavelength of 550 nm.

### Lactate dehydrogenase (LDH) assay

Following the applied treatments, cell death was examined by measuring LDH released to the medium, using a simple two-step LDH enzymatic reaction kit (Takara, Tokyo, Japan). Medium LDH contents were always normalized to total LDH contents^23^.

### TUNEL (terminal deoxynucleotidyl transferase dUTP nick end labeling) assay

Neuronal cells were seeded into the 96-well tissue-culture plates (at 3 × 10^4^ cells/cm^2^). Following OGD/R treatment, TUNEL In Situ Cell Death Detection Kit (Roche Diagnostics Co) was utilized to quantitatively examine cell apoptosis intensity. Cells were stained with TUNEL (Invitrogen, 5 μM). Cell nuclei were co-stained with DAPI and visualized through a fluorescent microscope (Leica, Shanghai, China). For each treatment, at least 500 cells in five random views (1 × 200 magnification) were counted to calculate TUNEL ratio (% vs. DAPI).

### Mitochondrial depolarization assay

In stressed/dying cells with mitochondrial depolarization, the fluorescence dye JC-1 will form green monomers by aggregating in the mitochondria^26^. Following the applied treatments, neuronal cells were stained with JC-1 (5 μg/mL, Sigma), washed and tested immediately using a fluorescence spectrofluorometer (F-7000, Hitachi, Japan) machine at test-wavelength of 545 nm (green). The JC-1 fluorescence images, integrating both the green (at 545 nm) and red (at 625 nm) wavelength were presented.

### Annexin V-FACS assay of apoptosis

Following the applied treatments, neuronal cells were stained with Annexin V and Propidium Iodide (PI), washed and tested immediately using a FACS machine (BD, Shanghai, China). Annexin V ratio was recorded.

### RNA-pull down assay

The pull-down assay was performed as described previously^27^. Briefly, the miR-422a (see sequence in ref. ^27^) and the mutant miR-422a (see sequence in ref. ^27^) single-stranded RNAs were labeled with biotin (at 5′), and then were individually transfected to cultured SH-SY5Y cells. The achieved lysates (using the described lysis buffer^27^) were pre-cleared and thereafter incubated with the beads coated with RNase-free BSA (Sigma) and yeast tRNA (Sigma)^27^. The beads were incubated and washed^27^. The bound RNAs were then analyzed by qPCR.

### Quantitative real-time PCR (qPCR)

Total RNA was extracted by Trizol reagents (Invitrogen, Shanghai, China) and quantified. For each condition, 500 ng of RNA was utilized for reverse transcription by SYBR Green SuperMix^28^. qPCR was performed by the 7900HT Fast Real-Time PCR system (Applied Biosystems). qPCR quantification was through 2^—ΔCt^ method using the following formula: 2^—(Ct of target gene—Ct of reference gene)^. The data presented were normalized to *GAPDH*. Expression of LncRNA D63785(Lnc-D63785) and miR-422a was normalized to *U6 RNA*. The primers of this study were listed in Table 1.

**Table-1:** Sequences utilized in this study.

qPCR primers	Forward (5′-3′)	Reverse (5′-3′)
LncRNA D63785	ACTGACGTATTTCTGGACCCAC	TTGCTGCTGACACGCCG
MiR-422a	ACTGGACTTAGGGTCAG	GAACATGTCTGCGTATCTC
U6	CTCGCTTCGGCAGCACAT	TTTGCGTGTCATCCTTGCG
GAPDH (Human)	CATGGGTGGAATCATATTGGAA	GAAGGTGAAGGTCGGAGT
MEF2D (Human)	GTTAGCGAGCAAGGGTCTGT	CCGCACACTGTTTCCAGAAC
MAPKK6 (Human)	GGCTACTTGGTGGACTCTGTTG	CATCGTGATGCCCAGACTCCAA
GAPDH (Mouse)	CATCACTGCCACCCAGAAGACTG	ATGCCAGTGAGCTTCCCGTTCAG
MEF2D (Mouse)	GGTTTCCGTGGCAACACCAAGT	GCAGGTGAACTGAAGGCTGGTA
MAPKK6 (Mouse)	TGGTGGAGAAGATGCGTCACGT	GTCACGGTGAATGGACAGTCCA
Oligonucleotides	5′-3′	
miR-422a mimic sequence	ACUGGACUUAGGGUCAGAAGGC	
mimic control sequence	UUCUCCGAACGUGUCACGUTT	
miR-422a inhibition sequence	GCCUUCUGACCCUAAGUCCAGU	
Inhibitor control sequence	UUCUCCGAACGUGUCACGUTT	

### Western blotting

Neuronal cells were seeded into the six-well tissue-culture plates at 70–80% confluence. The protein lysates (40 μg per treatment) were separated by 10–12.5% SDS-PAGE gels, then transferred to PVDF membranes (Millipore, Shanghai, China). Each PVDF membrane was blocked in PBST with 10% nonfat milk, thereafter incubated with the designated primary and secondary antibodies. ECL reagents (Roche, Shanghai, China) were added to detect signals under X-ray films, and Image J software (NIH) utilized to quantify the total gray of each protein band. For Western blotting, the same set of lysate samples were run in sister gels when necessary to test different proteins. The exact amount of protein lysates, 40 μg lysates per lane, were loaded in each lane.

### Forced overexpression of miR-422a

The pre-miR-422a (sequence, *GAGAGAAGCACUGGACUUAGGGUCAGAAGGCCUGAGUCUCUCUGCUGCAGAUGGGCUCUCUGUCCCUGAGCCAAGCUUUGUCCUCCCUGG*) was provided by Applied Biosystems (Shanghai, China), sub-cloned into the pSuper-puro-GFP construct (provided by Dr. Di^25^). The construct was transfected to HEK-293T cells together with the lentivirus package plasmids mix (psPAX2 and pMD2.G, Genechem), generating pre-miR-422a expression lentivirus (“lv-miR-422a”). The virus was collected, enriched and filtered, added to cultured SH-SY5Y cells (polybrene was added to the culture medium). After 24 h, SH-SY5Y cells were subjected to puromycin (0.5 μg/mL) selection for another 4–5 passages. Control cells were transduced with non-sense scramble control microRNA lentivirus (“miR-C”). In the stable neuronal cells, mature miR-422a (sequence, *ACUGGACUUAGGGUCAGAAGGC*) expression was tested by qPCR.

### miR-422a inhibition

The pre-miR-422a antisense (“antagomiR-422a”, purchased from Applied Biosystems) was annealed and sub-cloned into the GV248 lentiviral vector (Shanghai Genechem Co.). It was thereafter transfected with lentivirus package plasmids mix to HEK293T cells. The antagomiR-422a lentivirus (lv-antagomiR-422a) was added to cultured SH-SY5Y cells. After 24 h, the transfection medium was removed and replaced with fresh medium. Puromycin was then added to select stable cells for 4–5 passages. miR-422a expression was tested by qPCR in the stable cells.

### Lnc-D63785 overexpression

The sequence of Lnc-D63785^27^ was amplified by PCR with primers F (5′-*CGGAATTCTTGCTGCTGACACGCCGA*) and R (5′-*ACGCGTCGACACTGACGTATTTCTGGACCCACT*). An in vitro site-directed mutagenesis system was utilized to generate a miR-422a-binding mutant Lnc-D63785 (based on the sequence listed in ref. ^27^) by Genechem (Shanghai, China). The PCR products (WT- and Mut-) were ligated into the pSuper-puro-GFP (Addgene, Shanghai, China) construct, transfected to HEK-293T cells with the lentivirus plasmids mix (Genechem), generating Lnc-D63785-expression lentivirus (lv-Lnc-D63785, both WT- and Mut-). The lentivirus was then collected, enriched and filtered, and then added to SH-SY5Y cells for 48 h. Puromycin was then added to select the stable cells. Ectopic overexpression of Lnc-D63785 was confirmed by qPCR assays.

### Transfection of oligonucleotides

SH-SY5Y cells or the primary murine neurons were seeded into six-well plates at 60% confluence. Exact 100 pmol of scrambled negative control siRNA (“si-C”), Lnc-D63785 siRNA (two different sequences, “seq-1/-2”, see ref. ^27^), miR-422a mimic, the mimic control sequence, miR-422a inhibitor sequence or the inhibitor control sequence were transfected to SH-SY5Y cells or the murine neurons with Lipofectamine 2000, respectively. After 24 h, transfection was repeated one more round. Expression of target genes was verified by qPCR.

### N6-methyladenosine (m6A)-RNA immunoprecipitation and qPCR (MeRIP-qPCR)

The m6A antibody and the rabbit IgG^29^ were respectively conjugated to 20 μl Beads protein A/G mixed magnetic beads^29^. A 100 μg aliquot of fragmented total RNA was incubated with the antibody in immunoprecipitation buffer^29^ plus with 40U RNase inhibitor. RNA was eluted from the beads as described^29^. Following phenol extraction and ethanol precipitation, the m6A-enriched RNA was reversely transcribed and tested by qPCR assays, with Lnc-D63785 level normalized to Input controls.

### shRNA

The human METTL3 short hairpin RNA [two shRNAs, with two nonoverlapping sequences (“s1/s2”), as reported early^30,31^] was inserted into the GV369 construct (Genechem Co., Shanghai, China). The construct, along with the lentivirus package constructs, was transfected to HEK-293T cells for 24 h. The generated shRNA lentivirus was filtered, enriched, and added to cultured SH-SY5Y cells. Puromycin was then added to select the stable cells. Silencing of METTL3 was verified by Western blotting. Control cells were treated with the lentiviral scramble control shRNA (“shC”, Genechem, Shanghai, China).

### Statistical analysis

The investigators were blinded to the group allocation during all experiments. Experiments in this study were repeated at least three times. Data were expressed as mean ± standard deviation (SD). Statistics were analyzed by one-way analysis of variance (ANOVA) followed by a Scheffe’s *f*-test by the SPSS 21.0 software (SPSS Inc., Chicago, IL). To test significance between two treatment groups, a two-tailed unpaired *T* test (Excel 2007) was utilized. *p* < 0.05 was considered significant. All the protocols of this study were approved by Ethics Committee of Soochow University.

## Results

### OGD/R induces miR-422a elevation in neuronal cells

**Fig. 1:**
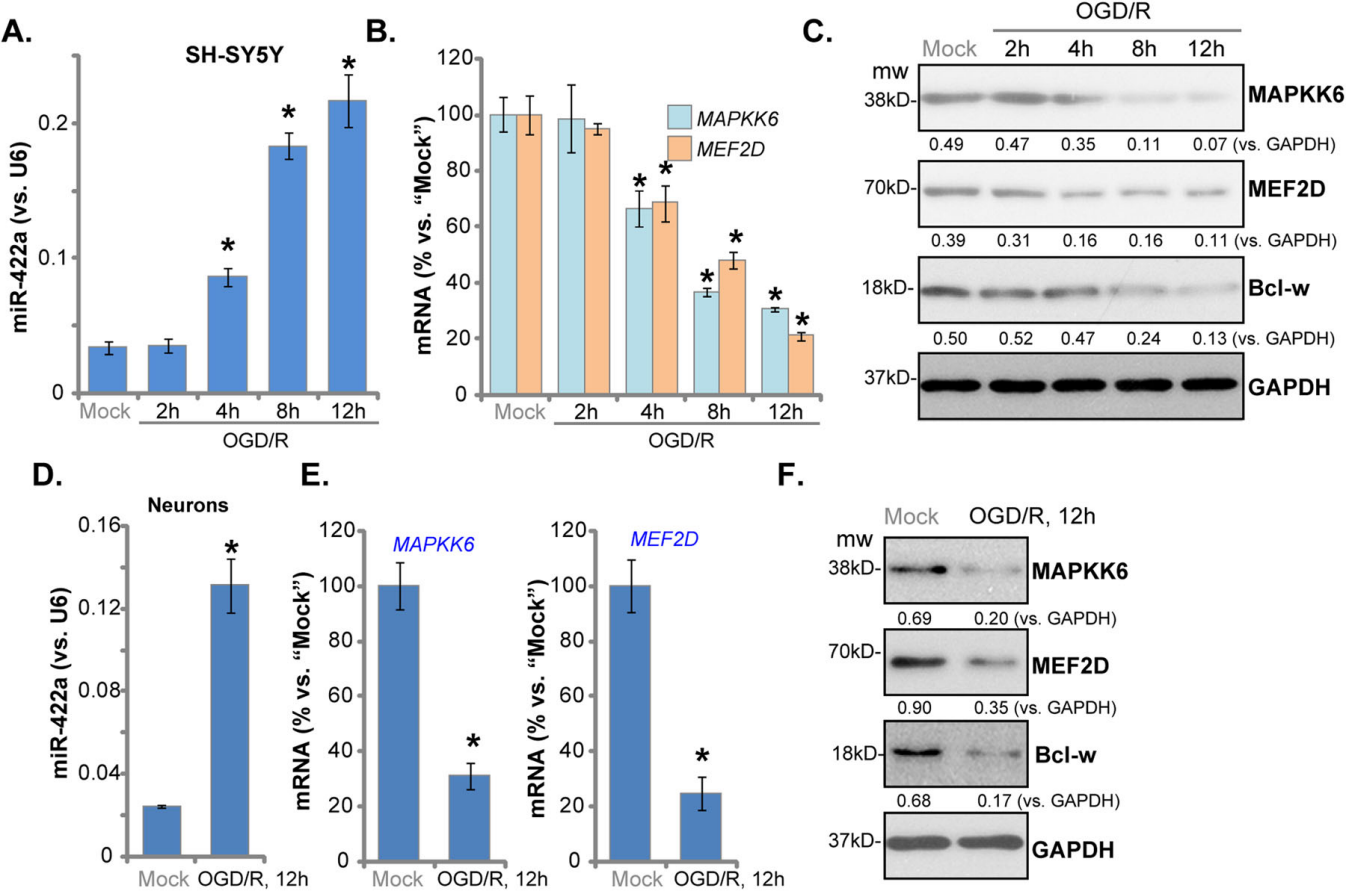
OGD/R induces miR-422a elevation in neuronal cells. SH-SY5Y cells (**a**–**c**) or primary murine cortical neurons (“neurons”, **d**–**f**) were maintained under oxygen glucose deprivation (OGD) for 4 h, followed by re-oxygenation (“OGD/R”) for the applied time; Expression of miR-422a and listed mRNAs was tested by qPCR assays (**a**, **b**, **d** and **e**); Listed proteins in total cell lysates were tested by Western blotting (**c** and **f**). The listed proteins were quantified and normalized to GAPDH (**c** and **f**). “Mock” stands for the mock treatment (norm-oxygenated medium with glucose) (Same for all Figures). Data indicate standard deviation (SD, *n* = 5). *p < 0.05 vs. “Mock” cells. Each experiment was repeated four times and similar results were obtained.

To test whether OGD/R can affect miR-422a expression, differentiated SH-SY5Y cells were exposed to oxygen glucose deprivation (OGD) for 4 h, as previously-described^24,25,32^. After OGD exposure, cells were maintained in regular medium (“re-oxygenation”, OGD/R) for various time periods. The qPCR assay results, Fig. 1a, demonstrated that miR-422a expression levels increased substantially in a time-dependent manner to 1.04 ± 0.15, 2.55 ± 0.19, 5.44 ± 0.29, and 6.42 ± 0.57 fold of the “Mock” control level, following 2, 4, 8, and 12 h of OGD/R (Fig. 1a).

Previous studies show that miR-422a acts as a tumor suppressor in cancer cells by targeting *mitogen-activated protein kinase kinase 6* (*MAPKK6*)^33^ and *myocyte enhancer factor-2D* (*MEF2D*)^27^. Examining the mRNA and protein levels of these verified miR-422a targets in SH-SY5Ycells, we found that *MAPKK6* and *MEF2D* mRNA (Fig. 1b) and protein (Fig. 1c) were significantly downregulated after OGD/R (Fig. 1b). Studies have shown that MEF2D is a key transcription factor required for neuronal survival^34^, essential for the expression of Bcl-w^35^, an anti-apoptotic Bcl-2 family protein in neurons^35^. We found that Bcl-w protein expression was also downregulated in OGD/R-stimulated SH-SY5Y cells (Fig. 1c).

In primary murine neurons, OGD/R procedure similarly induced miR-422a upregulation (5.38 ± 0.54 folds of control) (Fig. 1d). Consequently, *MAPKK6* and *MEF2D* mRNA (Fig. 1e) and protein (Fig. 1f) levels were downregulated, as well as Bcl-w protein expression (Fig. 1f). Collectively, OGD/R induced miR-422a elevation, leading to downregulation of its targets *MAPKK6* and *MEF2D* in neuronal cells.

### miR-422a inhibition attenuates OGD/R-induced neuronal cell death and apoptosis

**Fig. 2:**
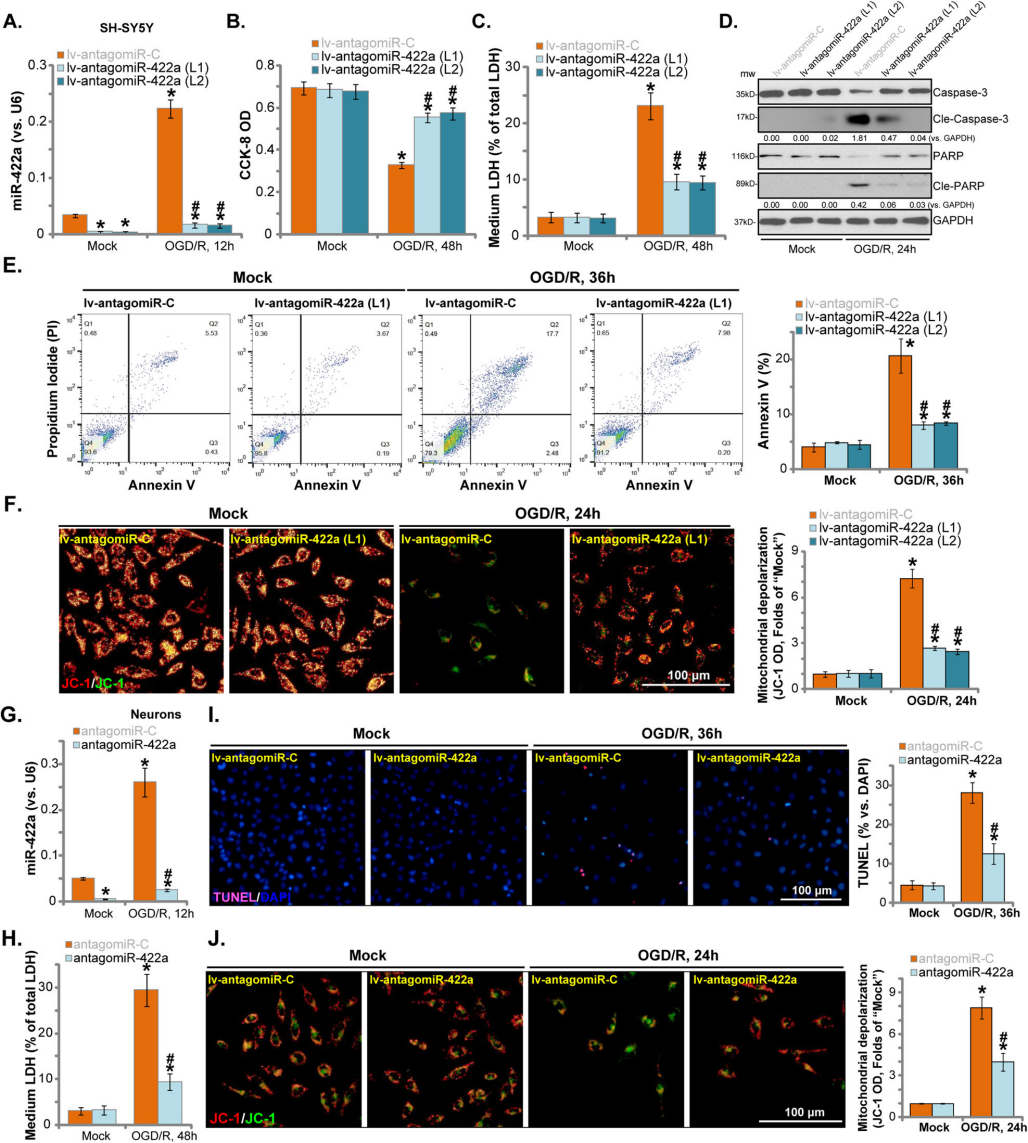
miR-422a inhibition attenuates OGD/R-induced neuronal cell death and apoptosis. SH-SY5Y cells were infected with pre-miR-422a anti-sense lentivirus (“lv-antagomiR-422a”) or non-sense miR inhibitor control lentivirus (“lv-antagomiR-C”) for 24 h, followed by puromycin selection (4–5 passages) to establish stable cells. Cells were maintained under oxygen glucose deprivation (OGD) for 4 h, followed by re-oxygenation (“OGD/R”) for the applied time; miR-422a expression (**a**), cell viability (**b**), cell death (**c**), cleaved-/or total-caspase-3/-PARP expression (**d**) and cell apoptosis (**e**) were tested by the assays mentioned in the text, with mitochondrial depolarization examined by JC-1 staining assay (**f**). The primary murine neurons were transfected with non-sense miR inhibitor control (“antagomiR-C”, 100 pmol × two rounds) or miR-422a inhibitor oligonucleotides (“antagomiR-422a”, 100 pmol × two rounds), followed by the same OGD/R procedure for applied time; miR-422a expression (**g**) and cell death (medium LDH release, **h**) were tested; Cell apoptosis and mitochondrial depolarization were tested by TUNEL staining (**i**) and JC-1 assay (**j**), respectively. Data indicate standard deviation (SD, *n* = 5). *p < 0.05 vs. “Mock” cells. ^**#**^*p* < 0.05 vs. OGD/R-treated “lv-antagomiR-C”/“antagomiR-C” neuronal cells. Each experiment was repeated three times and similar results were obtained. Bar = 100 μm (**f**, **i** and **j**).

To study the potential role of miR-422a in OGD/R-induced neuronal cytotoxicity, we inhibited miR-422a with a pre-miR-422a anti-sense lentivirus (“lv-antagomiR-422a”) transduced into SH-SY5Y cells. Following selection by puromycin, two stable SH-SY5Y cell lines (“L1/L2”) were established in which mature miR-422a levels were significantly downregulated (Fig. 2a), and OGD/R-induced miR-422a upregulation was completely blocked by lv-antagomiR-422a (Fig. 2a). Notably, the OGD/R-induced decrease in viability (CCK-8 OD) (Fig. 2b) and cell death (LDH medium release, Fig. 2c) were significantly attenuated by miR-422a inhibition.

To confirm the results of CCK-8 and LDH, cell apoptosis was examined. As shown, OGD/R treatment in SH-SY5Y cells induced significant apoptosis activation evidenced by cleavage of caspase-3-PARP [Poly (ADP-ribose) polymerases] (Fig. 2d), an increased Annexin V-positive staining (Fig. 2e). Importantly, miR-422a inhibition potently inhibited OGD/R-induced apoptosis activation in SH-SY5Y cells (Fig. 2d, e). Additional experimental results showed that OGD/R induced mitochondrial depolarization, causing accumulation of JC-1 green monomers fluorescence (Fig. 2f), which was attenuated with lv-antagomiR-422a (Fig. 2f). The lv-antagomiR-422a itself did not affect the function of SH-SY5Y cells (Fig. 2b–f).

In the primary murine neurons, transfection of the miR-422a inhibitor oligonucleotides (“antagomiR-422a”) blocked OGD/R-induced miR-422a upregulation (Fig. 2g), that attenuated OGD/R-induced neuronal death (LDH medium release, Fig. 2h), apoptosis activation (TUNEL staining assay, Fig. 2i), and mitochondrial depolarization (Fig. 2j). Thus, miR-422a inhibition can significantly attenuate OGD/R-induced neuronal cell death and apoptosis, indicating an essential function of miR-422a elevation in OGD/R-induced neuronal cell death and apoptosis.

### Forced overexpression of miR-422a induces neuronal cell death and apoptosis

**Fig. 3:**
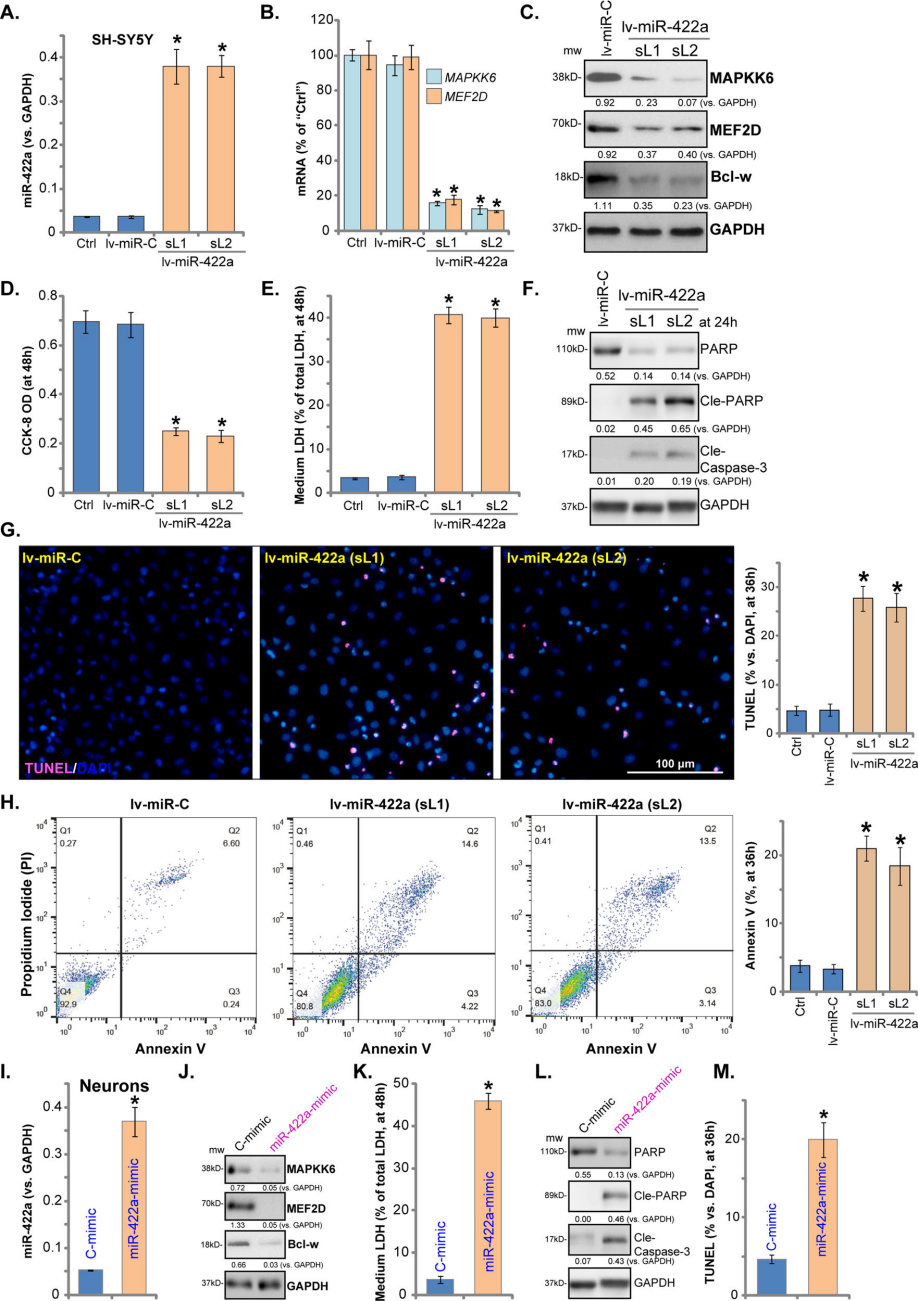
Forced overexpression of miR-422a induces neuronal cell death and apoptosis. SH-SY5Y cells were transduced with pre-miR-422a lentivirus (“lv-miR-422a”) or the miR control lentivirus (“lv-miR-C”), followed by puromycin selection, and the stable cells (“sL1” and “sL2”) were achieved. Expression of miR-422a (**a**), *MAPKK6* and *MEF2D* mRNAs (**b**) was tested by qPCR; MAPKK6, MEF2D and Bcl-w proteins were shown as well (**c**). Cells were further cultured for applied time, and then cell viability (**d**), cell death (**e**), expression of cleaved-caspase-3/-PARP (**e**) and cell apoptosis (**g**, **h**) were tested by the assays mentioned in the text. The primary murine neurons were transfected with miR-422a mimic (100 pmol) or the miR nonsense control mimic (“C-mimic”, 100 pmol) for 48 h, and then miR-422a expression (**i**), listed proteins expression (**j**), medium LDH contents (**k**), cleaved-caspase-3/-PARP levels (**l**) and cell apoptosis (**m**) were tested, with results quantified. Expression of listed proteins was quantified and normalized to GAPDH (**c**, **f**, **j** and **l**). “Ctrl” stands for the parental control cells. *p < 0.05 vs. “lv-miR-C”/“C-mimic” cells. Data indicate standard deviation (SD, *n* = 5). Each experiment was repeated three times and similar results were obtained. Bar = 100 μm (**g**).

Our data suggests that miR-422a elevation mediates OGD/R-induced neuronal cell death and apoptosis. Therefore, ectopic overexpression of miR-422a should mimic OGD/R-induced actions. To test this, two stable cell lines were established, “lv-miR-422a-sL1/sL2”, where miR-422a expression levels were increased over tenfold (vs. control cells, Fig. 3a). Consequently, miR-422a mRNA targets, *MAPKK6* and *MEF2D*, were significantly downregulated (Fig. 3b), and MAPKK6, MEF2D and Bcl-w protein expression reduced (Fig. 3c).

The forced overexpression of miR-422aresulted in a significant reduction in viability (CCK-8 OD) (Fig. 3d) and cell death (LDH release, Fig. 3e) in SH-SY5Y cells. Furthermore, lv-miR-422a induced apoptosis activation, as indicated by caspase-3-PARP cleavage (Fig. 3f), increased TUNEL-positive nuclei ratio (Fig. 3g) and Annexin V-positive cell ratio (Fig. 3h). The miR control lentivirus (“lv-miR-C”) had no significant effect on expression of miR-422a targets (Fig. 3a–c) and SH-SY5Y cell functions (Fig. 3d–h).

Testing the miR-422a mimic oligonucleotides (“miR-422a-mimic”) on primary murine neurons resulted in significant elevation of miR-422a (Fig. 3i) and silencing of its targets (MAPKK6, MEF2D and Bcl-w, Fig. 3j). Functionally, the miR-422a-mimic induced cell death (LDH release, Fig. 3k), caspase-3-PARP cleavage (Fig. 3l) and apoptosis activation (TUNEL-positive nuclei ratio increase, Fig. 3m).Therefore, forced overexpression of miR-422a can mimic OGD/R-induced actions and induce neuronal cell death.

### Decreased LncRNA D63785 expression is responsible for OGD/R-induced miR-422a accumulation and subsequent neuronal cell death and apoptosis

**Fig. 4:**
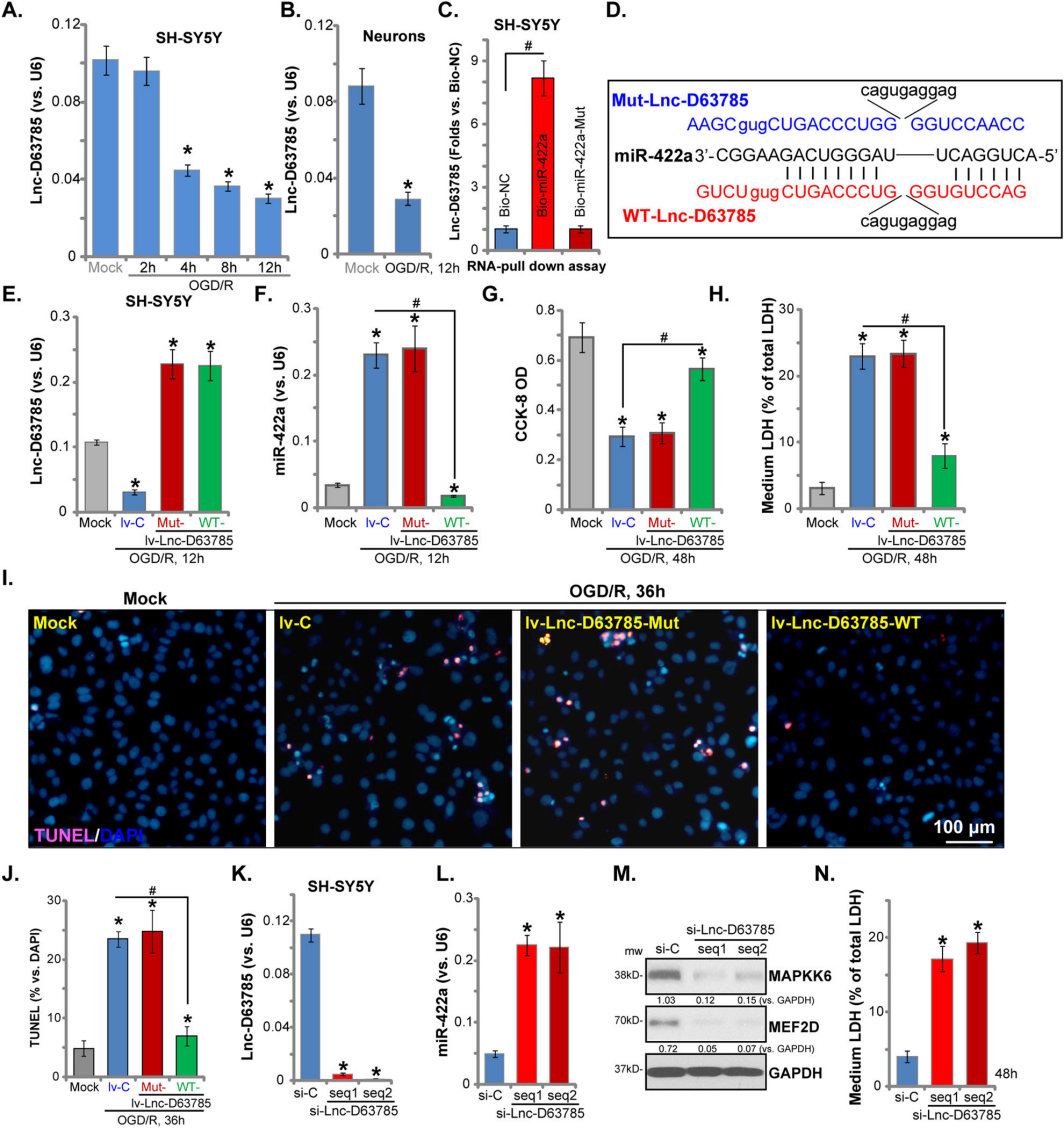
Decreased LncRNA D63785 expression is responsible for OGD/R-induced miR-422a accumulation and subsequent neuronal cell death and apoptosis. SH-SY5Y cells (**a**) or primary murine neurons (**b**) were maintained under oxygen glucose deprivation (OGD) for 4 h, followed by re-oxygenation (“OGD/R”) for the applied time; LncRNA D63785 (Lnc-D63785) expression was tested by qPCR assays. SH-SY5Y cells were transfected with biotinylated miR-422a (Bio-miR-422a) or its mutant form (Bio-miR-422a-Mut), and then a biotin-based pull-down assay was carried out to examine Lnc-D63785 expression, the latter was normalized to a biotinylated mimic control (Bio-NC) by qPCR assays (**c**); SH-SY5Y cells were transduced with the lentiviral constructs with wild-type (WT-) Lnc-D63785, miR-422a-binding mutant (“Mut-”) Lnc-D63785 (sequences listed in **d**), or the control vector (“lv-C”), and then stable cells were established via puromycin selection; Cells were subjected to OGD/R procedure for applied time, and then Lnc-D63785 and miR-422a expression was tested by qPCR assays (**e**, **f**); Cell viability (**g**) and death (**h**) were tested by CCK-8 and medium LDH release assays, respectively; Cell apoptosis was tested by TUNEL staining assay (**i**–**j**). SH-SY5Y cells were transfected with Lnc-D63785 siRNAs (“seq1/2”, with nonoverlapping sequences, 100 pmol) or scramble control nonsense siRNA (“si-C”, 100 pmol) for 48 h, and then Lnc-D63785 and miR-422a expression was tested by qPCR assays (**k**, **l**); Listed proteins were tested by Western blotting assay (**m**); Cell death (LDH release, **n**) was tested as well. Expression of listed proteins was quantified and normalized to GAPDH (**m**). Data indicate standard deviation (SD, *n* = 5). *p < 0.05 vs. “Mock” cells (**a**–**j**). *p < 0.05 vs. “si-C” cells (**k**–**o**). ^#^*p* < 0.05 (**c**, **f**–**j**). Each experiment was repeated three times and similar results were obtained. Bar = 100 μm (**i**).

The potential mechanism of OGD/R-induced miR-422a elevation was explored. Zhou et al., reported that LncRNA D63785 (Lnc-D63785) can function as a competitive endogenous RNA (ceRNA) of miR-422a. Lnc-D63785 blocks miR-422-dependent suppression of its target genes (i.e., *MEF2D*)^27^. OGD/R time-dependently decreased Lnc-D63785 expression in SH-SY5Y cells (Fig. 4a) and in OGD/R-treated primary murine neurons (Fig. 4b). To demonstrate that Lnc-D63785 directly binds miR-422a we employed a pull-down assay, finding that Lnc-D63785 was pulled down by biotinylated miR-422a in SH-SY5Y cells (Fig. 4c), but not by a mutant miR-422a^27^ (Fig. 4c).

To further examine the link between miR-422a accumulation and Lnc-D63785 reduction, lentiviral expression constructs, encoding the wild-type (WT-) Lnc-D63785 or the miR-422a-binding mutant (Mut-) Lnc-D63785 (see sequence in Fig. 4d)^27^, were individually transduced into SH-SY5Y cells. As shown the lentiviral construct (“lv-Lnc-D63785”) induced significant Lnc-D63785 (both WT- and Mut-) expression, even in OGD/R-treated cells (Fig. 4e). Significantly, OGD/R-induced miR-422a accumulation was completely blocked by overexpression of the WT-, but not the Mut-Lnc-D63785 (Fig. 4f).

Functional studies demonstrated that WT-Lnc-D63785 prevented the OGD/R-induced reduction of SH-SY5Y cell viability (Fig. 4g) and cell death (Fig. 4h), while the Mut-Lnc-D63785 was ineffective (Fig. 4g, h). In addition, OGD/R-induced apoptosis activation (TUNEL-positive nuclei ratio increasing, Fig. 4i) was decreased with overexpression of WT-Lnc-D63785, but not the mutant (Fig. 4i, j). These results support that decreased Lnc-D63785 expression is responsible for OGD/R-induced miR-422a accumulation and neuronal cell death.

Based on our results, Lnc-D63785 silencing is anticipated to induce neuronal cell death. To confirm this, SH-SY5Y cells were individually transfected with two Lnc-D63785 siRNAs (“seq1/2”, with nonoverlapping sequences^27^). Both resulted in Lnc-D63785 silencing (Fig. 4k), miR-422a accumulation (Fig. 4l) and depletion of its targets (MAPKK6 and MEF2D, Fig. 4m). As a result, Lnc-D63785 siRNA induced robust SH-SY5Y cell death (Fig. 4n). These results support a pivotal role for Lnc-D63785 downregulation in regulating OGD/R-induced miR-422a accumulation and neuronal cell death.

### OGD/R induces METTL3-dependent Lnc-D63785 m6A methylation in neuronal cells

**Fig. 5:**
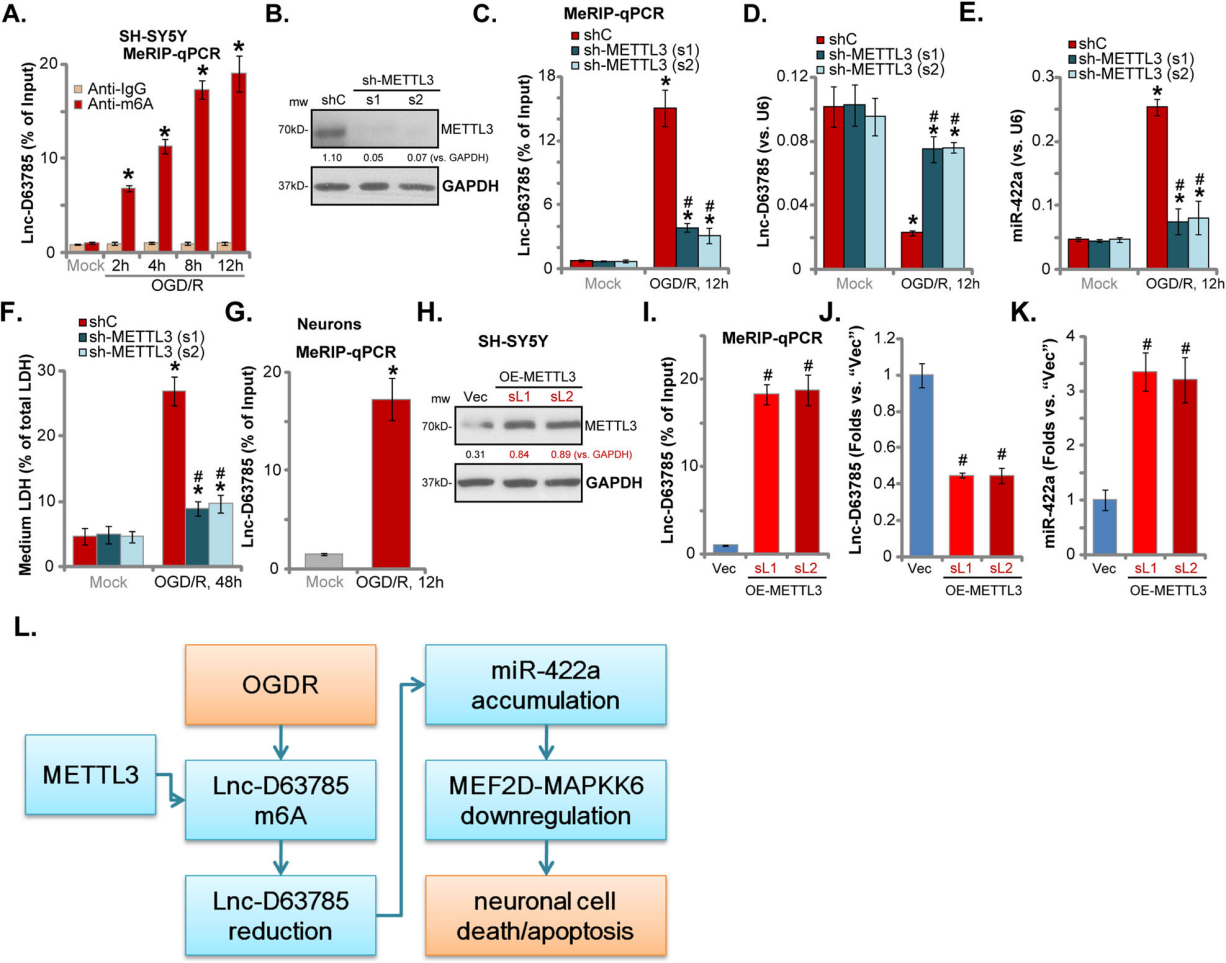
OGD/R induces METTL3-dependent Lnc-D63785 m6A methylation in neuronal cells. SH-SY5Y cells (**a**) or the primary neurons (**g**) were subjected to OGD/R stimulation, and then tested by MeRIP-qPCR assay of Lnc-D63785 m6A methylation. Expression of listed proteins in the stable SH-SY5Y cells with applied METTL3 shRNA (s1/s2, two different sequences) or scramble control shRNA (“shC”) was shown (**b**); Cells were subjected to OGD/R stimulation and then cultured for applied time periods, and relative Lnc-D63785 m6A methylation level was tested by MeRIP-qPCR assay (**c**); Expression of Lnc-D63785 (**d**) and miR-422a (**e**) was tested by qPCR assays, with cell death tested by LDH release assay (**f**). Stable SH-SY5Y cells expressing the lentiviral METTL3 expression construct (OE-METTL3-sL1/sL2, two lines) or the empty vector (“Vec”) were established, and then expression of METTL3 protein (**h**), Lnc-D63785 (**j**), and miR-422a (**k**) was tested by Western blotting and qPCR assays; The relative Lnc-D63785 m6A methylation level was tested by MeRIP-qPCR assay (**i**); **l** The proposed signaling cascade of this study. Expression of listed proteins was quantified and normalized to GAPDH (**b** and **h**). Data indicate standard deviation (SD, *n* = 5). *p < 0.05 vs. “Mock” cells. ^**#**^*p* < 0.05 vs. OGD/R-treated “shC” cells (**c**–**f**). ^**#**^ *p* < 0.05 vs. “Vec” cells (**i**–**k**). Each experiment was repeated three times and similar results were obtained.

To test the possible mechanism of Lnc-D63785 reduction we examined N6-methyladenosine (m6A) methylation of Lnc-D63785. N6-methyladenosine (m6A) methylation has been shown to regulate the degradation of non-coding RNAs, including LncRNAs^36^. MeRIP-qPCR results demonstrated that, following OGD/R stimulation, Lnc-D63785 m6A methylation levels were significantly increased in SH-SY5Y cells (Fig. 5a). M6A modifications occur via m6A methyltransferases such as methyltransferase-like 3 (METTL3)^18^.

To demonstrate that METTL3 is responsible for Lnc-D63785 M6A modifications, lentiviral METTL3 shRNAs (s1/s2, two different sequences^31^) were individually transduced into SH-SY5Y cells, resulting in robust METTL3 downregulation (Fig. 5b). In SH-SY5Y cells, the two applied METTL3 shRNAs potently inhibited Lnc-D63785 m6A methylation (Fig. 5c). Importantly, OGD/R-induced reduction of Lnc-D63785 was reversed by METTL3 silencing (Fig. 5d), suggesting that m6A methylation is the primary cause of Lnc-D63785 reduction in OGD/R-treated neuronal cells. Significantly, OGD/R-induced miR-422a accumulation was inhibited in METTL3-silenced SH-SY5Y cells (Fig. 5e) and cell death (Fig. 5f) attenuated. In the primary murine neurons, OGD/R similarly induced Lnc-D63785 m6A methylation (Fig. 5g).

To examine the effect of METTL3 overexpression, a lentiviral METTL3 expression construct was utilized to create stable SH-SY5Y cells, and as shown, METTL3 protein expression was elevated in the OE-METTL3 SH-SY5Y cells (Fig. 5h). As a result, Lnc-D63785 m6A methylation was increased (Fig. 5i), whereas its expression was downregulated (Fig. 5j), and miR-422a accumulation detected (Fig. 5k). Based on these results, we propose that OGD/R induces METTL3-dependent Lnc-D63785 m6A methylation, causing a reduction of Lnc-D63785R, miR-422a accumulation, leading to neuronal cell death and apoptosis (Fig. 5l).

## Discussion

There is an urgent need to identify novel biomarkers for early diagnosis and prognostic evaluation of ischemic stroke. Recent studies have explored the potential of abnormally-expressed circulating miRNAs as a diagnostic or prognostic biomarker for this disease^14,15^. It has been reported that circulating miR-422a, a brain-enriched miR, is significantly upregulated in patients with acute ischemic stroke^14^. Our results show that OGD/R induces significant miR-422a accumulation, mediating neuronal cell death and apoptosis. In SH-SY5Y cells and primary murine cortical neurons, miR-422a inhibition, by antagnomiR-422a, significantly attenuated OGD/R-induced viability reduction, cell death and apoptosis. Conversely, ectopic miR-422a overexpression mimicked OGD/R-induced actions, inducing neuronal cytotoxicity. Therefore, miR-422a accumulation may play a key role in the mechanism of OGD/R-induced neuronal cell injury.

The transcription factor MEF2D is an important neuronal survival factor^37–39^, promoting neuronal survival by dictating expression of Bcl-w^37–39^. Conversely, MEF2D hyper-phosphorylation, downregulation or degradation can cause neuronal cell death^37–39^. In SH-SY5Y cells and primary murine neurons, we found that the upregulation of miR-422a by OGD/R led to downregulation of its target MEF2D (also Bcl-2), which could explain the subsequent neuronal cell death and apoptosis. Furthermore, another important miR-422a target, MAPKK6 (a key upstream of p38^40^), was also downregulated in OGD/R-treated cells. Forced overexpression of miR-422a, by lv-miR-422a, decreased MEF2D-MAPKK6 expression in neuronal cells.

LncRNAs are a class of non-coding RNAs with the length over 200 nt, generally considered to be mRNA-like transcripts^41,42^. LncRNA can function as ceRNAs to sponge target miRNAs, thereby antagonizing miRNA-induced activity^41,42^. The study by Zhou et al., has shown that Lnc-D63785 is a key ceRNA of miR-422a^27^. The results of the current study suggest that the OGD/R-induced reduction of Lnc-D63785 is the primary cause of miR-422a accumulation and neuronal cytotoxicity. Lnc-D63785 directly associates with miR-422a in the neuronal cells. Restoring Lnc-D63785 expression, by a lentiviral construct, not only abolished OGD/R-induced miR-422a accumulation, but also attenuated neuronal cell death. Conversely, overexpression of amiR-422a-binding mutant Lnc-D63785 was unable to affect OGD/R-induced neuronal cell death/apoptosis. Importantly, siRNA-mediated knockdown of Lnc-D63785 induced miR-422a accumulation and neuronal cell injury, mimicking the neuronal cytotoxicity induced by OGD/R. Overall these results support that decreased Lnc-D63785 expression is responsible for OGD/R-induced miR-422a accumulation and subsequent neuronal cell death.

Emerging studies have proposed that m6A methylation is an important regulatory mechanism for the expression, function, and stabilization of LncRNA transcripts in human cells^20–22^. LncRNA m6A modifications may also regulate gene expression^20–22^. In the present study, we found that OGD/R stimulation led to METTL3-mediated Lnc-D63785 m6A methylation, which is the primary cause of Lnc-D63785 downregulation. METTL3 silencing, by targeted shRNAs, reversed OGD/R-induced Lnc-D63785 m6A methylation and downregulation as well as miR-422a accumulation in SH-SY5Y cells. Furthermore, OGD/R-induced cytotoxicity was also potently attenuated by METTL3 shRNA. Conversely, forced overexpression of METTL3 in SH-SY5Y cells led to Lnc-D63785 m6A methylation, reduced Lnc-D63785 expression and miR-422a accumulation. Therefore, METTL3-induced Lnc-D63785 m6A methylation is one key mechanism of OGD/R-induced miR-422a accumulation and subsequent neuronal cell death.

## Conclusions

We conclude that OGD/R leads to Lnc-D63785 m6A modification and decreased expression, consequently resulting in miR-422a accumulation, downregulation of its targets (*MEF2D*-*MAPKK6*), and neuronal cell death/apoptosis.
